# Interictal cognitive event-related potentials in migraine subtypes: association with neuropsychiatric symptoms

**DOI:** 10.3389/fneur.2026.1875362

**Published:** 2026-07-29

**Authors:** Marta Waliszewska-Prosół, Błażej Misiak, Igor Petrušić, Karol Marschollek, Sławomir Budrewicz

**Affiliations:** 1 Department of Neurology, Wroclaw Medical University, Wroclaw, Poland; 2 Department of Psychiatry, Wroclaw Medical University, Wroclaw, Poland; 3 Faculty of Physical Chemistry, Laboratory for Advanced Analysis of Neuroimages, University of Belgrade, Belgrade, Serbia

**Keywords:** anxiety, aura, burden, CERP, sleep

## Abstract

**Background:**

Subjective cognitive decline is a common phenomenon among migraine patients. This study aimed to compare cognitive event-related potentials (CERP) across different migraine subtypes during the interictal phase and to evaluate their relationship with clinical and neuropsychiatric parameters.

**Methods:**

A study was conducted involving 173 participants: 55 with migraine without aura (MwoA), 40 with migraine with aura (MwA), 23 with migraine aura without headache (MAwH), and 55 healthy controls (CG). CERP analysis included N200 and P300 latencies and amplitudes. Disease burden and comorbidities were assessed using the MIDAS, HIT-6, the Insomnia Severity Index (ISI), and the Hospital Anxiety and Depression Scale (HADS). All patients underwent brain MRI.

**Results:**

MwoA patients exhibited significantly prolonged N200 and P300 latencies alongside decreased P300 amplitudes compared to the CG. In contrast, while both MwA and MAwH patients also showed prolonged latencies across CERP components, with MwA demonstrating the longest latencies among all subtypes, their P300 amplitudes did not differ significantly from healthy controls. Notably, the MAwH group demonstrated significantly higher N200 and P300 amplitudes when compared directly to the MwoA group, achieving the highest overall values among the migraine subtypes. MwA demonstrated the longest latencies among all subtypes. Correlation analysis revealed that P300 latencies in MwoA were linked to attack frequency, while in MwA they correlated with disability scores (HIT-6, MIDAS). Notably, in the MAwH group, P300 parameters showed strong positive correlations with anxiety, depression, and insomnia scores. Across all subtypes, progressive latency prolongation was associated with longer disease duration.

**Conclusion:**

Our findings confirm significant interictal disturbances in neuronal networks involved in cognitive processing. Furthermore, the strong correlation between CERP parameters and neuropsychiatric symptoms in MAwH suggests that psychological comorbidities are strongly linked to the modulation of neurophysiological responses in this specific subtype.

## 1 Introduction

Migraine is a complex neurological disorder frequently associated with cognitive and affective comorbidities (1, 2). Although patients often report “*brain fog*,” memory lapses, and impaired focus or executive functioning, these symptoms remain overlooked in clinical practice (3–5). Notably, these cognitive disturbances can persist during the interictal phase, significantly affecting patients’ daily functioning between attacks (6). While the exact etiology of these subjective difficulties remains unknown, it is hypothesized that they may be modulated by underlying neurotransmitter imbalances, such as alterations in the serotonergic or glutamatergic systems (7).

Event-related potentials (ERPs) offer an objective, non-invasive method to assess the neural networks involved in information processing (8). As a measure of cognitive integrity, ERPs allow for the evaluation of memory, decision-making, and attentional focus (8, 9). The P300 component, or “*attention wave*,” is the primary marker used to assess stimulus processing time (latency) and the allocation of cognitive resources (amplitude) (9–11). Complementing this, the N200 wave reflects early stimulus identification and the brain’s “*readiness*” to engage in a task (9, 10).

However, previous electrophysiological studies in migraine face significant limitations that obscure a full understanding of interictal cognitive functioning. Most historical ERP research has either treated migraine as a monolithic entity or pooled all aura patients into a single heterogeneous group, largely ignoring the distinct neurobiological profiles of specific subtypes, such as migraine aura without headache (MAwH) (10, 11). Furthermore, while cognitive “*brain fog*” and psychological distress frequently co-occur in these patients, existing literature has rarely integrated objective electrophysiological markers with standardized measures of affective burden and sleep quality across well-defined subgroups (12). This leaves a critical knowledge gap regarding whether interictal cognitive processing is driven primarily by pain frequency or by underlying neuropsychiatric comorbidities, particularly in patients who experience the neurological symptoms of aura without the subsequent nociceptive headache phase (13–15).

Despite the clinical relevance of cognitive dysfunction, comparative data across specific migraine subtypes remain limited. The aim of this study was to evaluate brain bioelectrical activity during the interictal phase in patients with different migraine subtypes using ERPs, and to correlate these findings with disease progression as well as levels of anxiety, depression, and insomnia.

## 2 Materials and methods

### 2.1 Patients and clinical assessment

Participants were recruited between December 2022 and March 2025 from the Headache Center at the Department of Neurology, Wroclaw Medical University, Poland. The study included 118 patients (74% female, mean age 36.3 years) with episodic migraine who met the diagnostic criteria of the International Classification of Headache Disorders, 3rd edition (ICHD-3) (16).

Clinical and neurophysiological evaluations were performed during the interictal phase, defined as at least 48 h after and at least 48 h before a migraine attack. Patients were required to abstain from acute/pain medication for a minimum of 3 days prior to the recording.

The study protocol consisted of a detailed neurological examination, cognitive screening using the Montreal Cognitive Assessment (MoCA) and the Symbol Digit Modalities Test (SDMT), and a brain MRI.

Cognitive and executive functions were further evaluated using the Clock Drawing Test (CDT) and the Trail Making Test Part A (TMT-A). The CDT was employed as a brief screening tool to assess visuospatial and constructional abilities, as well as executive functioning. The TMT-A was utilized primarily to evaluate visual scanning, processing speed, and psychomotor performance, where the total time to complete the task (in seconds) served as the primary outcome measure.

Migraine characteristics were assessed using the following metrics: monthly migraine days (MMD), monthly headache days (MHD), the Headache Impact Test-6 (HIT-6), and the Migraine Disability Assessment (MIDAS). Psychological distress and sleep quality were measured using: (1) the Hospital Anxiety and Depression Scale (HADS) and (2) the Insomnia Severity Index (ISI).

Exclusion criteria were:

1. Presence of any neurological, psychiatric, systemic, cognitive, or sleep disorders.
2. Other co-occurring primary or secondary headaches.
3. History of traumatic brain injury.
4. Hearing defects.
5. Prophylactic migraine treatment in the last 3 months.
6. History of chronic migraine or medication overuse headache.
7. Presence of any other pain syndromes.
8. Administration of stimulants or medications affecting brain bioelectrical activity (e.g., steroids, neuroleptics, antiepileptics, antidepressants, sedatives, or hypnotics).
9. Underweight (BMI < 18.5 kg/m^2^) or obesity (BMI ≥ 35 kg/m^2^).
10. Pregnancy and lactation.
11. Active malignancy.

The control group (CG) consisted of 55 healthy volunteers matched for age, sex, and education level (40 women, 15 men; mean age 35.4 years). The exclusion criteria and study protocol were identical for the CG.

### 2.2 Event related potentials protocol

A detailed description of the recording methods was provided in our previous publication (17, 18). The procedure complied with the recommendations of the International Federation of Clinical Neurophysiology (IFCN) (15, 19). Briefly, ERPs were recorded using surface Ag/AgCl electrodes placed at Fz, Cz, and Pz according to the international 10–20 system, referenced to linked earlobes with a forearm ground.

Responses were recorded using a Nicolet 1,000 Viking system, with a 0.3–70 Hz bandpass filter, a sweep time of 1,000 ms, and a pre-stimulus baseline of 250 ms. Impedance was maintained below 5 kΩ. ERPs were elicited using a classic auditory “oddball paradigm.” Target tones (2000 Hz) occurred in 20% of trials, and non-target tones (1,000 Hz) in 80%. Stimuli were presented at 70 dB intensity with a 200 ms duration. At least 30 target trials were averaged per run, with two runs performed per patient. The N200 and P300 components were identified as the most negative and positive peaks within the 180–320 ms and 280–450 ms latency windows, respectively. Peak-to-peak amplitudes and latencies were analyzed.

### 2.3 Ethics statement

Approval for this study was given by the Bioethics Committee at Wroclaw Medical University (number of permission: KB-952/2022). Informed consent was obtained from each participant prior to study inclusion.

### 2.4 Statistical analysis

Bivariate differences between migraine patients and healthy controls were analyzed using the chi-squared test (categorical variables), the Mann–Whitney U test (non-normally distributed continuous variables), and t-tests (normally distributed continuous variables). Normality was assessed using the Kolmogorov–Smirnov test. Analysis of covariance (ANCOVA) was performed to test differences in ERP parameters after adjusting for age and education. Before ANCOVA, ERP parameters were transformed into z-scores. Partial correlations, controlling for age and sex, were calculated to analyze associations between continuous variables. Results were corrected for multiple comparisons using the false discovery rate (FDR) (*p* < 0.05). Data analyses were conducted using SPSS software (version 28).

## 3 Results

### 3.1 Clinical characteristics

The general characteristics of the study population are presented in Table 1. No significant between-group differences were found regarding age, sex, BMI, or education level. Based on the diagnostic criteria, 55 patients were diagnosed with migraine without aura (MwoA), 40 with migraine with aura (MwA), and 23 with migraine aura without headache (MAwH).

**Table 1:** Clinical characteristics of the sample.

	MwoA (*n* = 55)	MwA (*n* = 40)	MAwH (*n* = 23)	Control group (*n* = 55)
Sex (female/male)	40/15	30/10	17/6	40/15
Age (years)	35.1 ± 5.5	36.1 ± 5.8	38.5 ± 7.1	35.4 ± 7.7
Education (secondary/higher)	16/39	11/29	6/17	16/39
Disease onset (age)	18.1 ± 4	18.8 ± 3.8	19.5 ± 4	-
Disease duration (years)	16.9 ± 6.8	17.3 ± 8	18.9 ± 8.4	-
BMI (kg/m^2^)	24.5 ± 3.2	24.7 ± 3.4	24.2 ± 4	24.6 ± 3.1
MHD	8.04 ± 2.5	8.75 ± 2.6	-	-
MMD	6.6 ± 2.6	7.1 ± 2.4	4.9 ± 2.1	-
HIT-6 (score)	54.4 ± 8.7	57.4 ± 10.1	-	-
MIDAS (score)	29.4 ± 14.8	30.4 ± 13.9	-	-
HADS-A	6.38 ± 2.7	7.1 ± 2.9	9.7 ± 2.9	-
HADS-D	6.9 ± 2.8	7.4 ± 2.5	7.3 ± 2.7	-
ISI	6.25 ± 4	7.5 ± 3.9	8.1 ± 4.4	-

Data presented as mean ± SD or *n*. BMI, body mass index; CDT, Clock Drawing Test; HADS, Hospital Anxiety and Depression Scale; HIT-6, Headache Impact Test-6; ISI, Insomnia Severity Index; MAwH, migraine aura without headache; MHD, monthly headache days; MIDAS, Migraine Disability Assessment; MMD, monthly migraine days; MwA, migraine with aura; MwoA, migraine without aura.

Neurological examinations and cognitive screening results (MoCA, SDMT, CDT, and TMT-A) were within normal limits for all participants. Brain magnetic resonance imaging (MRI) showed no significant abnormalities in any of the patients.

### 3.2 Event-related potentials

The mean values and standard deviations of N200 and P300 parameters for all groups are summarized in Table 2.

**Table 2:** Mean values and standard deviations (SD) of N200 and P300 parameters in different types of migraine patients and in the control group (CG).

ERP	Mean ± SD				*p*-values					
	MwoA (*n* = 55)	MwA (*n* = 40)	MAwH (*n* = 23)	CG (*n* = 55)	MwoA vs CG	MwA vs CG	MAwH vs CG	MwoA vs MwA	MwoA vs MAwH	MwA vs MAwH
Latency (ms)										
N200 Fz	223.3 (32.0)	248.7 (37.6)	231.4 (31.0)	209.0 (20.9)	0.098	**<0.001**	**0.003**	**0.001**	0.603	0.989
Cz	224.0 (32.2)	248.9 (37.2)	234.1 (31.3)	208.1 (20.7)	0.100	**≤0.05**	0.223	**0.003**	**0.001**	1.000
Pz	225.1 (31.3)	249.7 (37.0)	238.2 (30.9)	208.9 (19.1)	0.635	**≤0.05**	**0.05**	**<0.001**	**0.001**	1.000
P300 Fz	330.2 (26.4)	347.8 (18.4)	334.4 (16.1)	307.2 (17.2)	**<0.001**	**<0.001**	**<0.001**	**0.001**	1.000	0.165
Cz	334.9 (23.8)	351.2 (17.6)	336.0 (17.3)	312.1 (21.5)	**<0.001**	**<0.001**	**0.001**	**0.001**	1.000	0.068
Pz	337.9 (24.7)	352.3 (19.3)	338.0 (8.4)	314.8 (26.1)	**<0.001**	**<0.001**	**0.001**	**0.005**	1.000	0.154
Amplitude (μV)										
N200 Fz	3.28 (1.64)	4.89 (2.24)	5.35 (1.96)	4.51 (2.83)	0.078	**<0.001**	**0.001**	**<0.001**	0.913	1.000
Cz	2.90 (1.44)	4.64 (2.06)	4.80 (1.76)	3.84 (3.01)	**0.040**	**<0.001**	**<0.001**	**0.001**	0.339	1.000
Pz	2.61 (1.26)	4.21 (2.31)	4.74 (2.53)	3.09 (2.24)	**0.031**	**<0.001**	**<0.001**	**0.002**	0.118	1.000
P300 Fz	5.86 (2.56)	8.18 (3.44)	9.38 (3.50)	7.37 (3.42)	0.091	1.000	0.106	**0.007**	**<0.001**	0.914
Cz	5.41 (2.15)	7.92 (3.39)	8.58 (3.34)	7.05 (2.97)	**0.015**	1.000	0.280	**0.001**	**<0.001**	1.000
Pz	4.90 (2.59)	7.26 (3.65)	8.36 (2.56)	7.00 (2.99)	**0.017**	1.000	0.067	**0.007**	**<0.001**	0.277

CG, control group; MwoA, migraine without aura; MwA, migraine with aura; MAwH, migraine aura without headache; SD, standard deviation; Fz, frontal region; Cz, central region; Pz, parietal region.

Statistically significant changes (*p* < 0.05). Significant *p* values are bolded.

#### 3.2.1 Latencies of N200 and P300

Significant differences in ERP latencies were observed between migraine patients and the control group (CG). P300 latencies at all electrode sites (Fz, Cz, Pz) were significantly prolonged in all three migraine subgroups (MwoA, MwA, and MAwH) compared to healthy controls (*p* < 0.001). Regarding the N200 component, the MwA and MAwH groups showed significantly longer latencies at the Fz and Pz locations compared to the CG. Patients with migraine with aura (MwA) exhibited the most pronounced prolongations; they demonstrated significantly longer P300 latencies across all locations and a longer N200 latency at the Fz site compared to both the CG and MwoA groups (*p* < 0.01). For the N200 component at the Cz and Pz locations, the latency prolongations in the MwA group compared to the CG were significant at the *p* ≤ 0.05 level. No significant differences in latencies were found between the MwA and MAwH groups.

#### 3.2.2 Amplitudes of N200 and P300

The analysis of ERP amplitudes revealed distinct patterns among migraine subtypes. P300 amplitudes were significantly lower in the MwoA group compared to the control group at the Cz (*p* = 0.015) and Pz (*p* = 0.017) sites. In contrast, P300 amplitudes in the MwA and MAwH groups did not differ significantly from the healthy controls.

For the N200 component, patients with MwA and MAwH exhibited significantly higher amplitudes across all electrode sites compared to the CG (*p* < 0.003). Conversely, MwoA patients showed significantly lower N200 amplitudes at the Cz and Pz locations compared to controls (*p* < 0.05). Notably, the MAwH group demonstrated the highest N200 and P300 amplitudes among all migraine subtypes, showing significant differences when compared to the MwoA group (*p* < 0.001).

### 3.3 Electrophysiological correlations with clinical and psychological parameters

**Figure 1:**
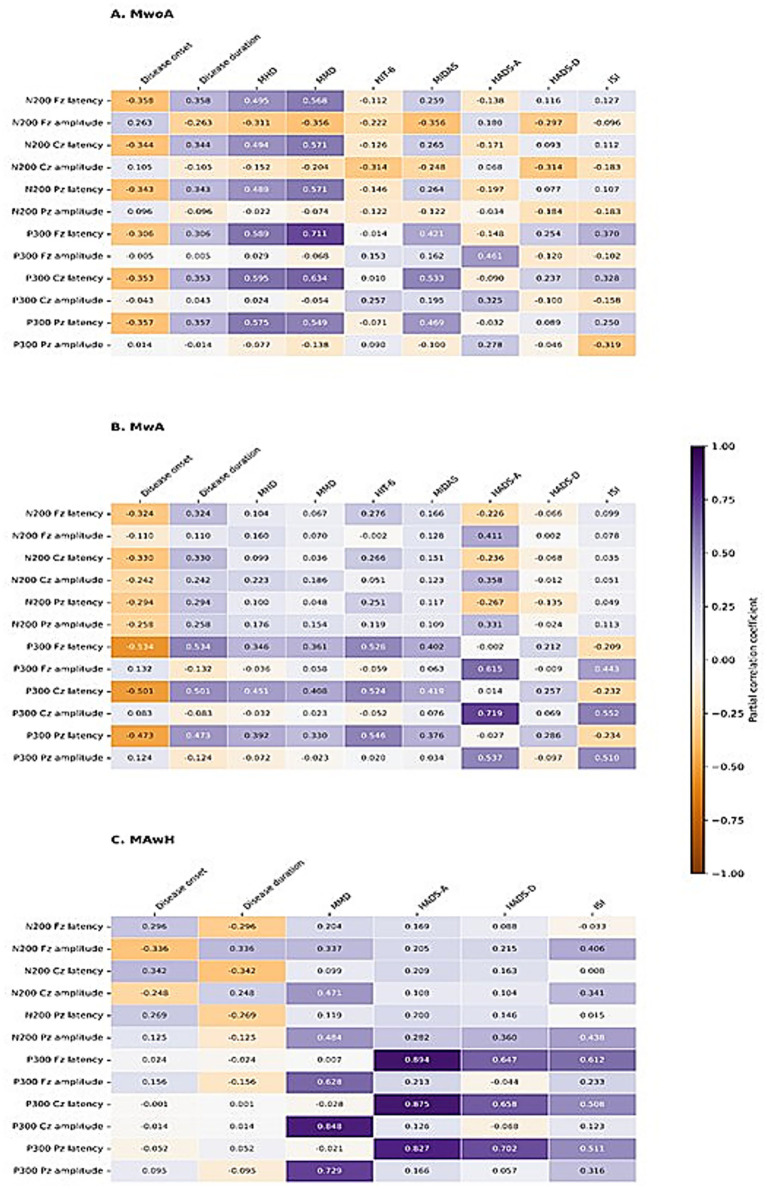
Heatmaps of partial correlation coefficients between ERP parameters and clinical characteristics across the study groups: **(A)** patients with migraine without aura (MwoA), **(B)** patients with migraine with aura (MwA), and **(C)** patients with migraine aura without headache (MAwH). All correlations were age- and sex-adjusted. Positive correlations are represented by purple shades, and negative correlations by orange-brown shades.

Statistical analysis revealed distinct correlation patterns across the three groups studied, as illustrated in the heatmaps presented in Figure 1.

In the MwoA patients, prolonged latencies of the P300 component (Fz, Cz, and Pz) correlated positively with headache frequency, including MHD (*r* = 0.575 to 0.589, *p* < 0.05) and MMD (*r* = 0.549 to 0.711, *p* < 0.05) (Figure 1A). In contrast, the MwA group exhibited significant positive correlations between P300 latencies and disability scores, particularly HIT-6 (*r* = 0.524 to 0.546, *p* < 0.05) and MIDAS (*r* = 0.376 to 0.419, *p* < 0.05) (Figure 1B).

In the MAwH group (Figure 1C), P300 latencies at Fz, Cz, and Pz locations demonstrated strong positive correlations with neuropsychiatric measures, including anxiety (HADS-A; *r* = 0.827 to 0.894, *p* < 0.05), depression (HADS-D; *r* = 0.647 to 0.702, *p* < 0.05), and insomnia severity (ISI; *r* = 0.508 to 0.612, *p* < 0.05). Additionally, P300 amplitudes in the MAwH group showed a strong positive correlation with the frequency of attacks (MMD; *r* = 0.628 to 0.848, *p* < 0.05).

Across all groups, latencies of the N200 and P300 components were negatively correlated with the age of disease onset (e.g., P300 Fz latency in MwA: *r* = −0.534, *p* < 0.05) and positively correlated with disease duration (e.g., P300 Fz latency in MwA: *r* = 0.534, *p* < 0.05).

### 3.4 Anxiety levels across migraine subtypes

**Figure 2:**
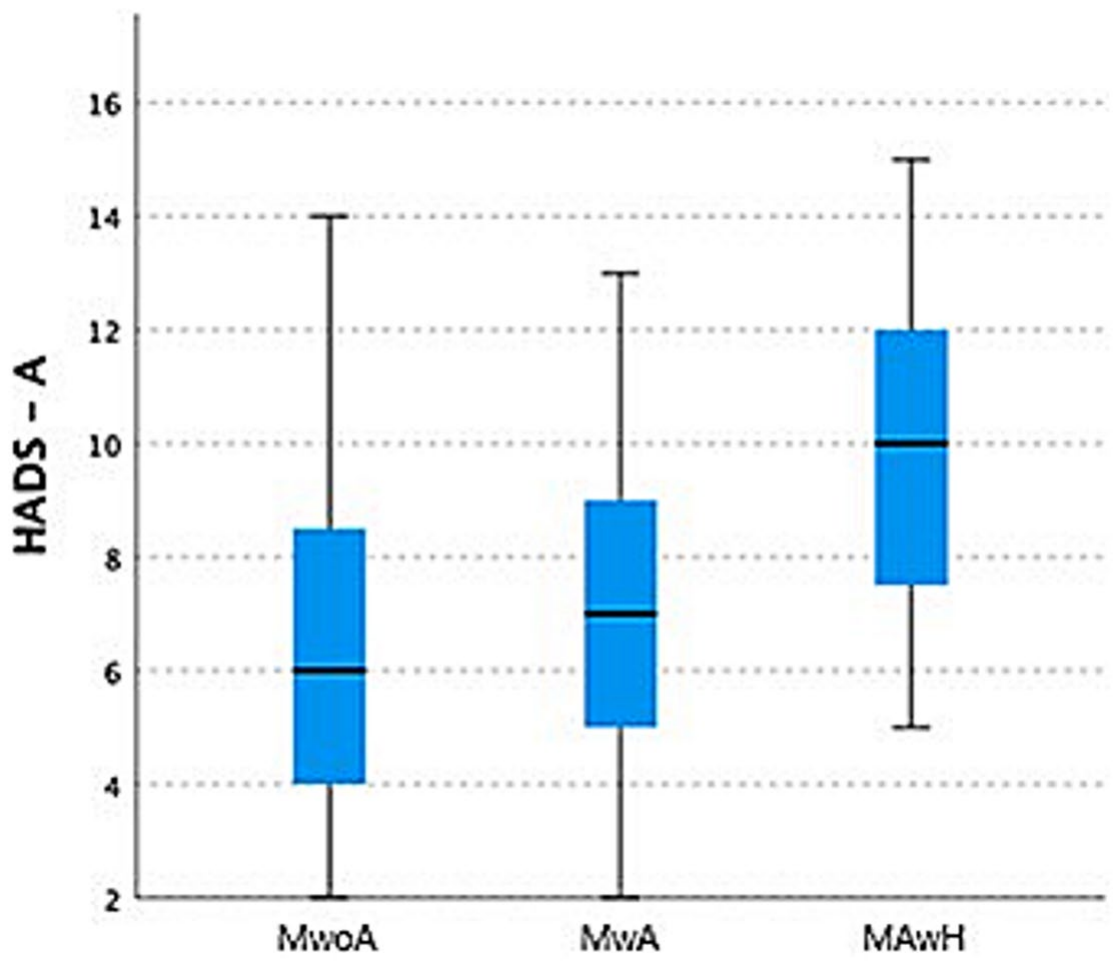
HADS-A scores by migraine subtype.

Anxiety levels (HADS-A) showed a progressive increase across the studied groups. Median scores were lowest in the MwoA group (6), followed by the MwA group (7), and reached the highest levels in the MAwH group (10) (Figure 2). The distribution indicates that patients with MAwH experience a significantly higher affective burden compared to other subtypes, with median scores reaching the clinical threshold for anxiety symptoms.

## 4 Discussion

Our study demonstrates that interictal cognitive processing is significantly altered across all migraine subtypes, though the nature of these changes varies. The MwoA group exhibited the “classic” neurophysiological profile of cognitive slowing: prolonged P300 latencies and reduced amplitudes. These findings align with previous studies suggest that recurrent pain leads to a depletion of attentional resources and slower stimulus evaluation (12, 20). Specifically, our observation of prolonged P300 latencies in MwoA (typically around 330–350 ms) is consistent with findings reported in other studies (21).

In contrast, patients with MwA and MAwH displayed a different pattern: while their latencies were the most prolonged (indicating slower processing speed), their P300 and N200 amplitudes were significantly higher than those of both the control group and MwoA patients. This phenomenon likely reflects cortical hyperexcitability, a well-documented trait in migraine with aura (22, 23). The increased amplitude may represent a compensatory mechanism or a lack of habituation to repetitive stimuli, where the brain over-allocates resources to process sensory information (24). Our results contrast with some earlier studies, such as those by Drake et al., who reported reduced P300 amplitudes in migraine patients (25). However, our findings of elevated amplitudes in aura subtypes (reaching 8–10 μV in the MAwH group) support the hypothesis that aura-related mechanisms fundamentally alter cortical reactivity differently than pain alone. This hypothesis is even more supported by the finding that there is an association between the complexity of MwA attack and prolongation of P300 latency (11).

The most striking finding of our study is the exceptionally strong correlation between P300 parameters and neuropsychiatric scores (HADS and ISI), specifically in the MAwH group. While MwoA patients’ results were driven by headache frequency, the MAwH group’s neurophysiological profile was dictated by affective burden. The integration of electrophysiological measures with anxiety and insomnia scales represents a novel approach, as few studies have explored these interdependencies across migraine subtypes. Our findings in the MAwH group align with the research of Valeriani et al. (13), who demonstrated a significant correlation between abnormal brain excitability and emotional symptomatology in migraine patients in a pediatric population. Specifically, we found that the correlation between P300 parameters and affective burden reached an exceptionally high range (*r* = 0.827 to 0.894), suggesting that in the absence of a headache phase, the interictal neurophysiological profile is almost entirely driven by the patient’s neuropsychiatric state.

Regarding sleep quality and affective states, our results align with the theoretical framework proposed by Rains, where comorbid insomnia in headache patients may exert a cumulative burden on the central nervous system, reflected in the slowing of cognitive processing (26). In our MAwH and MwA cohorts, poor sleep quality was associated with prolonged P300 latencies. It is possible that the absence of a headache phase in MAwH contributes to a state of constant sensory anticipation or cortical hyper-vigilance. According to the Attentional Control Theory, high anxiety can lead to heightened arousal while diverting neural resources toward emotional regulation. In this unique clinical model, the lack of subsequent trigeminovascular activation, which normally concludes a typical migraine attack, might hypothetically leave the cortex in a state of unresolved hyperexcitability (27). We hypothesize that this state could be mediated by shared, underlying serotonergic and glutamatergic dysregulation, suggesting that in the MAwH subtype, the neuropsychiatric profile may act as a key modulator of the interictal neurophysiological state. However, since our study is correlational, these biochemical and pathophysiological mechanisms remain purely speculative and require prospective verification (28–30).

The progressive prolongation of latencies associated with disease duration across all groups suggests that migraine is a progressive neurobiological disorder (31). Over years of attacks, the cumulative effect of “biochemical stress”-likely involving glutamatergic excitotoxicity, may lead to permanent changes in neuronal network efficiency (32, 33). Our results suggest that cognitive “brain fog” is not a monolithic symptom. In MwoA, it is a consequence of pain frequency, whereas in MwA and MAwH, it is deeply intertwined with anxiety and sleep quality. For these patients, addressing neuropsychiatric comorbidities may be just as important as traditional prophylactic treatment for improving cognitive “clarity.”

The primary strength of this study is the comprehensive characterization of the MAwH group, which is rarely analyzed as a distinct entity in neurophysiological research. By including three different migraine subtypes, we were able to demonstrate that cognitive processing in migraine is not uniform but heterogeneous, depending significantly on the presence of aura and the degree of neuropsychiatric burden.

Despite its clinical relevance, this study has several limitations. First, the sample size of the MAwH group, while sufficient for statistical significance, was relatively modest compared to the MwoA and MwA groups, reflecting the lower clinical prevalence of this subtype. Second, the cross-sectional design prevents us from establishing causal relationships; we cannot definitively determine whether neurotransmitter imbalances cause both anxiety and ERP changes, or if chronic anxiety leads to the observed cortical hyperexcitability (30, 33). Third, while we controlled for the interictal phase based on patient self-reports, we did not use electronic diaries to verify the exact timing of subsequent attacks prospectively, which may introduce a degree of recall bias. Fourth, it must be acknowledged that event-related potentials are inherently non-specific; similar abnormalities in P300 latency and amplitude have been documented in various other neurological and psychiatric disorders, potentially limiting the direct diagnostic specificity of these findings to migraine alone (17, 34–36).

## 5 Conclusion

Our study demonstrates that migraine subtypes exhibit distinct interictal neurophysiological profiles. In MwoA, cognitive resource depletion and slowed processing are primarily driven by headache frequency. Conversely, patients with aura, particularly MAwH, display persistent cortical hyperexcitability, where neuropsychiatric comorbidities (anxiety, depression, and insomnia) act as integral modulators of brain activity rather than mere secondary symptoms. These findings advocate for a personalized clinical approach: while pain management remains paramount in MwoA, addressing psychological burden and sleep quality is critical for mitigating cognitive impairment in aura subtypes.

## 6 Clinical implications

1. Cognitive impairment in migraine is not uniform; while patients without aura (MwoA) primarily demonstrate cognitive resource depletion, individuals with aura subtypes exhibit distinct cortical hyperexcitability alongside slowed information processing.
2. In patients experiencing migraine aura without headache (MAwH), neurophysiological dysfunction is profoundly driven by affective and sleep burdens, such as anxiety and insomnia, rather than pain frequency alone.
3. The cumulative effect of repeated migraine attacks over time contributes to a progressive neurobiological decline, visibly reflected in the gradual slowing of cognitive processing speed across all subtypes.
4. Effective management demands a personalized clinical approach; therapeutic efforts should focus on reducing attack frequency in MwoA, whereas mitigating psychological burden and improving sleep quality are critical to restoring cognitive clarity in aura subtypes.

## Data Availability

The raw data supporting the conclusions of this article will be made available by the authors, without undue reservation.
